# Intramural metastasis to the appendix from ascending colon cancer: a case report

**DOI:** 10.1186/s40792-020-00829-6

**Published:** 2020-04-10

**Authors:** Toshiya Abe, Hiroshi Sakai, Masataka Hayashi, So Nakamura, Shin Takesue, Masafumi Sada, Shingo Kozono, Yoshiki Kitaura, Yoshitaka Tanabe, Kazuyoshi Nishihara, Mari Mine, Sadafumi Tamiya, Toru Nakano

**Affiliations:** 1 Department of Surgery, Kitakyushu Municipal Medical Center, 2-1-1 Bashaku, Kokurakita-ku, Kitakyushu, 802-0077 Japan; 2 Department of Pathology, Kitakyushu Municipal Medical Center, Kitakyushu, Japan

**Keywords:** Intramural metastasis, Appendix, Colon cancer, Laparoscopic right hemi-colectomy

## Abstract

**Background:**

Intramural metastasis is rare in colorectal cancer, especially metastasis of ascending colon cancer to the appendix.

**Case presentation:**

A 64-year-old man was admitted to our hospital for surgery for ascending colon cancer detected by medical examination. Colonoscopy identified a type-2 tumor in the ascending colon, which was diagnosed as adenocarcinoma. Abdominal computed tomography revealed focal thickening of the ascending colon and middle of the appendix and swelling of the lymph nodes around the ileocolic artery. The patient underwent laparoscopic right hemi-colectomy with D3 lymph node dissection. Histopathological findings revealed that the ascending colon cancer was moderately differentiated adenocarcinoma with lymphatic and vascular invasion (stage IIIB; pT3N2M0). Additionally, moderately differentiated adenocarcinoma was observed mainly in the submucosa and muscularis propria of the appendix, which was approximately 10 cm proximal to the ascending colon cancer. These findings indicated intramural metastasis to the appendix from the ascending colon cancer. The patient experienced recurrence with lung metastasis 2.5 years after the first surgery.

**Conclusions:**

Intramural metastasis of ascending colon cancer to the appendix is extremely rare. Because the risk of recurrence and the prognosis for intramural metastasis has not been clarified, careful follow-up is recommended.

## Background

Intramural metastasis is rare in colorectal cancer, although this type of metastasis has been frequently reported in esophageal and gastric cancer [1, 2]. The presence of intramural metastasis is an important poor prognostic indicator [3]. We report an extremely rare case of intramural metastasis from ascending colon cancer to the appendix.

## Case presentation

**Fig. 1:**
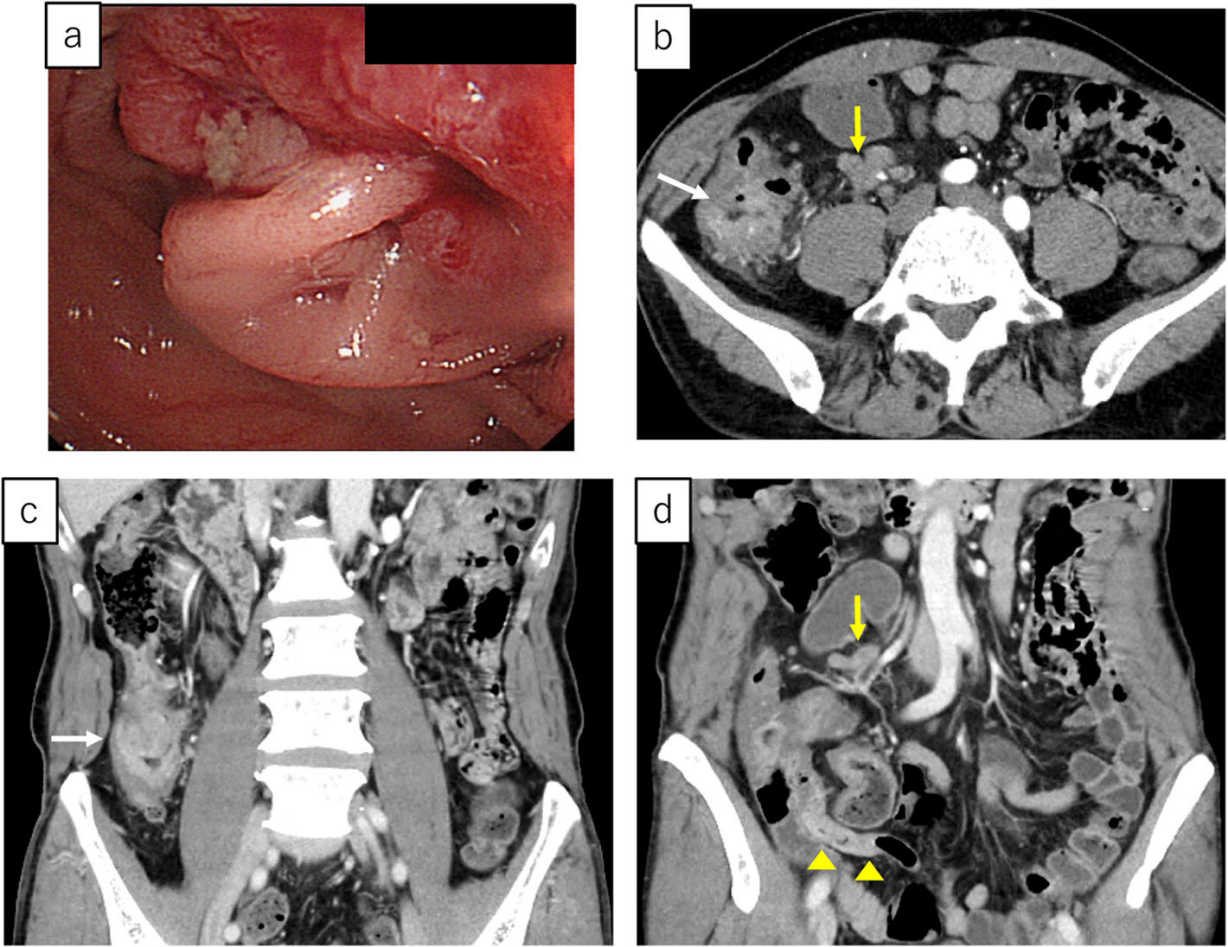
**a** Colonoscopy image showing a type-2 tumor in the ascending colon. **b–d** Enhanced abdominal computed tomography images showing focal thickening in the ascending colon (white arrow) and middle of the appendix (yellow arrowheads) and swelling of the lymph nodes around the ileocolic artery (yellow arrow)

**Fig. 2:**
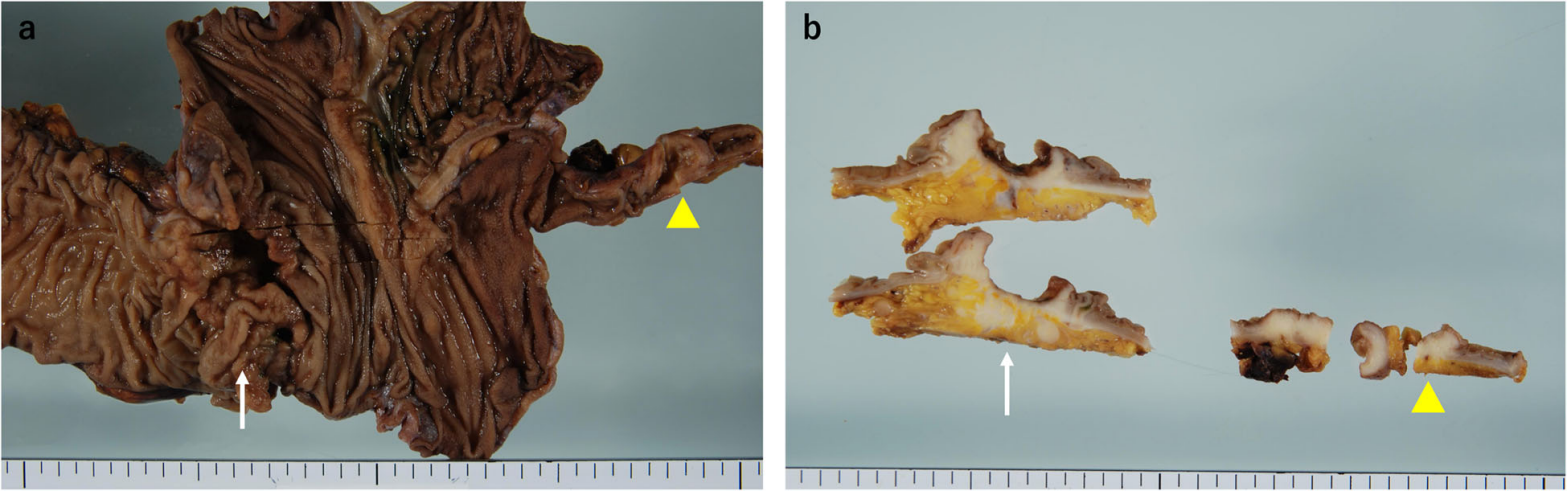
**a** Macroscopic examination of the resected specimens showing a circumferential type-2 lesion in the middle of the ascending colon and thickening of the middle of the appendix. **b** Gross appearance of a cross-section showing the primary tumor (white arrow) in the ascending colon and intramural metastasis in the appendix (yellow arrowhead)

**Fig. 3:**
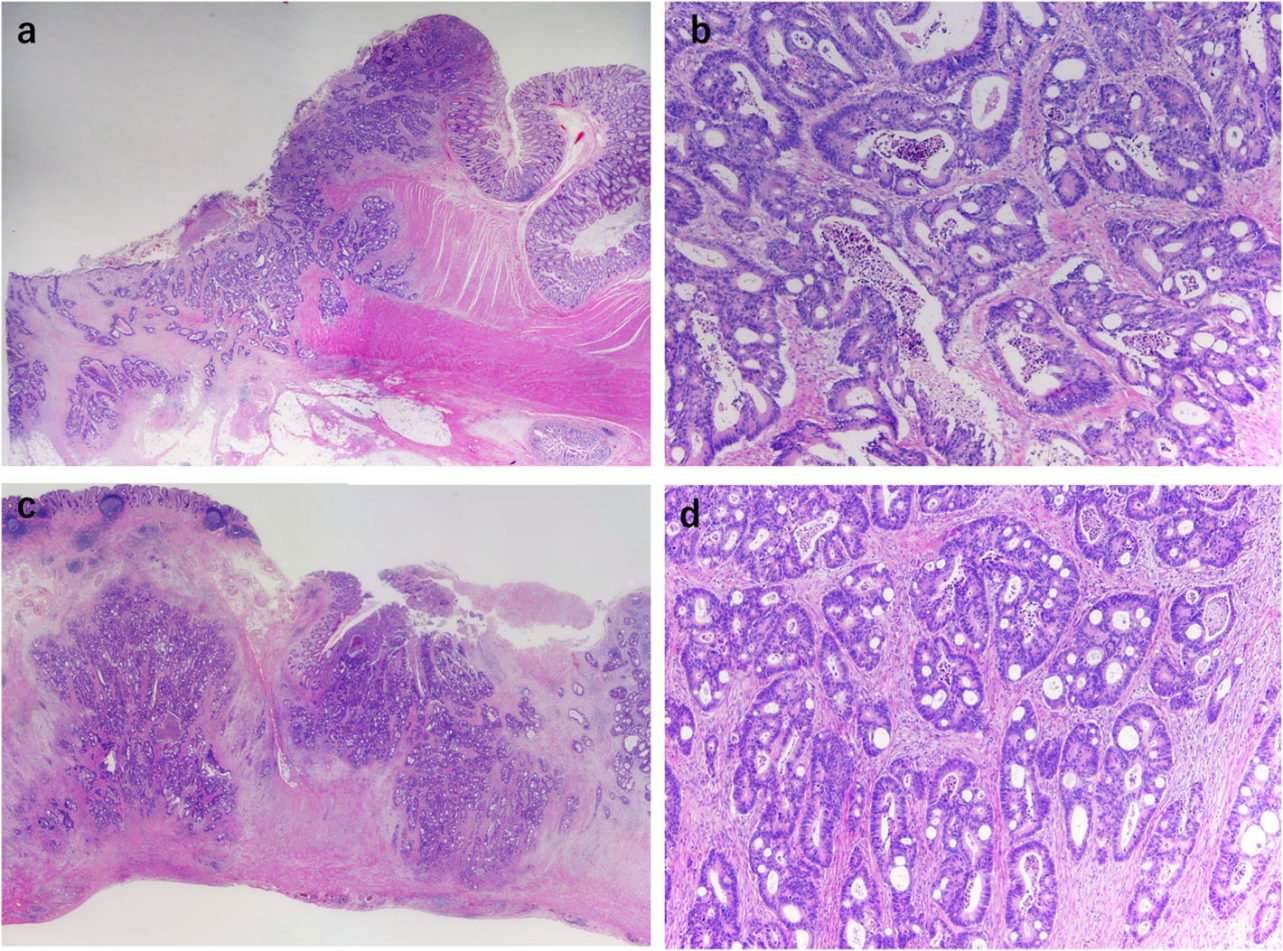
Histopathological findings (hematoxylin and eosin). **a** The mucosa between the primary ascending colon cancer and the appendix is normal (×1). **b** The primary colon cancer was moderately differentiated adenocarcinoma (×4). **c** Cancer cells are seen mainly in the submucosa and muscularis propria of the appendix. **d** Moderately differentiated adenocarcinoma cells similar to those seen in the ascending colon cancer specimen

**Fig. 4:**
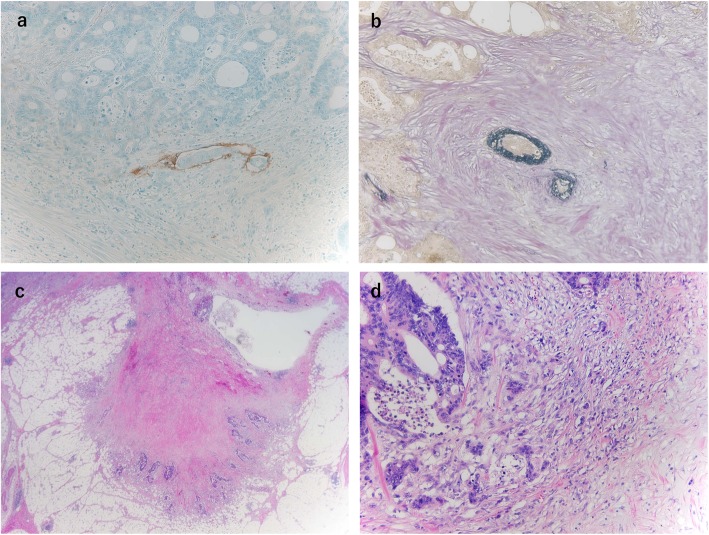
Histopathological findings. **a** Cancer cells are observed in the lymphatic vessels (×10) (D2-40). **b** Cancer cells are observed in the venous duct (×10) (EVG). **c** Extramural tumor deposits without lymph node structure are seen in the extramural adipose tissue (×20) (hematoxylin and eosin). **d** Tumor budding grade 2 is seen in the leading edge of the cancer cells (×2) (hematoxylin and eosin)

A 64-year-old man was admitted to our hospital for surgery for ascending colon cancer detected by colonoscopy to address the symptom of frequent diarrhea. The patient had no history of malignancy, and laboratory findings, including tumor marker levels, were within normal ranges. Colonoscopy identified a type-2 tumor in the ascending colon, which was diagnosed as adenocarcinoma by biopsy (Fig. 1a). Additionally, barium enema examination showed an irregular stenosis in the ascending colon. Subsequent abdominal computed tomography revealed focal thickening of the ascending colon and middle of the appendix and swelling of the lymph nodes around the ileocolic artery (Fig. 1b–d). Under a preoperative diagnosis of ascending colon cancer and tumor of the appendix, laparoscopic right hemi-colectomy with D3 lymph node dissection was performed. The macroscopic examination of the resected specimens revealed a circumferential type-2 lesion in the middle of the ascending colon and thickening of the middle of the appendix (Fig. 2). The mucosa between the ascending colon cancer and appendix was normal. Histopathological findings revealed that the ascending colon cancer was moderately differentiated adenocarcinoma with lymphatic and vascular invasion (stage IIIB; pT3N2M0, ly1, v1) (Fig. 3a, b and Fig. 4a, b). Extramural tumor deposits without lymph node structure were seen in the extramural adipose tissue (Fig. 4c). Tumor budding grade 2 was also seen in the leading edge of the cancer cells (Fig. 4d). In addition, moderately differentiated adenocarcinoma cells were observed in the submucosa and muscularis propria of the appendix (Fig. 3c, d), which was approximately 10 cm proximal to the ascending colon cancer. The tissue between the ascending colon cancer and the middle of the appendix was free of malignant cells. These findings indicated intramural metastasis to the appendix from the ascending colon cancer. The patient experienced the postoperative complication of chyle leak, which was treated by drainage, and he was discharged on postoperative day 23. The patient underwent neoadjuvant chemotherapy for 6 months after surgery; however, he developed lung metastasis 2.5 years after the first surgery and underwent segmentectomy of the upper lobe of the left lung.

## Discussion

Intramural metastasis is defined as a metastatic tumor spreading from the primary tumor to the digestive tract through the intramural lymphatic system [1]. Intramural metastasis is rare in colorectal cancer, although this type of metastasis has been frequently reported in esophageal and gastric cancer [1, 2]. To the best of our knowledge, there is only one report of suspected intramural metastasis from colon cancer to the appendix [4]. Regarding the mechanism of intramural metastasis, Li et al. [4] suggested that the absence of a right colic artery might be a cause in colon cancer metastasis to the appendix. However, because our patient had a right colic artery, this mechanism does not explain the intramural metastasis. Because lymphatic blockage develops when marked lymph node metastasis occurs along the artery, lymph flow might change to a retrograde direction, which might cause intramural metastasis [5]. In our patient, histopathological findings revealed lymphatic invasion and a high number of metastatic lymph nodes (8/16), mainly along the ileocolic artery. These findings might have caused the metastasis from the ascending colon cancer to the appendix, secondary to lymphatic blockage.

Recently, tumor-stroma interaction including extramural tumor deposits without lymph node structure or tumor budding has been discussed as the mechanism of metastasis and poor prognosis [6, 7]. In fact, both extramural tumor deposits and tumor budding were confirmed in our case although the relationship between intramural metastasis and these factors is unknown. Therefore, further studies are needed to determine the mechanism of intramural metastasis.

The distance of the intramural metastasis is usually within 2 cm in rectal cancer [5]. In our patient, the distance of the intramural metastasis was 10 cm compared with 5 cm in a previous similar report [4], and both distances were longer distances compared with intramural metastasis in rectal cancer. These distances are not a problem when we perform right hemi-colectomy for ascending colon cancer because the appendix is removed. However, there is a possibility of intramural metastasis from transverse colon cancer to the appendix; therefore, awareness of our rare case of intramural metastasis is important. Intramural metastasis is considered an important factor predicting poor prognosis in esophageal [3, 8, 9], gastric [2], and rectal [5] cancers. Although there is a lack of consensus regarding treatment strategies, intramural metastasis might also be a poor prognostic factor in metastasis from colon cancer to the appendix. In fact, our patient experienced recurrence with lung metastasis 2.5 years after the first surgery although the risk factor for lung metastasis might be lymph node metastasis. Adjuvant chemotherapy is recommended in the cases with lymph node metastasis (stage III) regardless of intramural metastasis [10]. Because the lymphatic invasion of the submucosal layer is one of the strong causes of intramural metastasis, there are some cases without lymph node metastasis [5]. Nakagoe et al. also showed that distal intramural spread is an independent risk factor, separate from the stage, for distant metastasis and poor prognosis in patients with rectal cancer [5]. In ESMO guidelines, it is recommended that only patients with stage II colorectal cancer who had high risk underwent adjuvant chemotherapy [11]. Therefore, these findings indicate that intramural metastasis might be an indication to perform adjuvant chemotherapy as high risk. Further studies are needed to determine the treatment strategies for colon cancer with intramural metastasis.

## Conclusions

We report an extremely rare case of intramural metastasis from ascending colon cancer to the appendix. Because recurrence and prognosis for intramural metastasis in colorectal cancer has not been clarified, careful follow-up is recommended.s
