# Correction to: Intramural metastasis to the appendix from ascending colon cancer: a case report

**DOI:** 10.1186/s40792-020-00844-7

**Published:** 2020-04-20

**Authors:** Toshiya Abe, Hiroshi Sakai, Masataka Hayashi, So Nakamura, Shin Takesue, Masafumi Sada, Shingo Kozono, Yoshiki Kitaura, Yoshitaka Tanabe, Kazuyoshi Nishihara, Mari Mine, Sadafumi Tamiya, Toru Nakano

**Affiliations:** 1 Department of Surgery, Kitakyushu Municipal Medical Center, 2–1-1 Bashaku, Kokurakita-ku, Kitakyushu, 802–0077 Japan; 2 Department of Pathology, Kitakyushu Municipal Medical Center, Kitakyushu, Japan

**Correction to: Surgical Case Reports**

**https://doi.org/10.1186/s40792-020-00829-6**

In the original publication of this article [1], an author’s name should be changed from Shin Takasue to Shin Takesue. The original article has been updated.
